# Assessing and addressing COVID-19 information needs via a weather application

**DOI:** 10.1093/jamiaopen/ooac016

**Published:** 2022-03-26

**Authors:** Mollie M McKillop, Courtney VanHouten, Carol Bales, Winnie Felix, Rezzan Hekmat, Gretchen Purcell Jackson

**Affiliations:** 1 Center for AI Research and Evaluation, IBM Watson Health, Cambridge, Massachusetts, USA; 2 Consumer Insights, The Weather Company, Atlanta, Georgia, USA; 3 Vanderbilt University Medical Center, Vanderbilt University, Nashville, Tennessee, USA

**Keywords:** COVID-19, consumer health informatics, public health, consumer health information needs, weather

## Abstract

We describe implementation and usage of a coronavirus disease 2019 (COVID-19) digital information hub delivered through the widely adopted The Weather Company (TWC) application and explore COVID-19 knowledge, behaviors, and information needs of users. TWC deployed the tool, which displayed local case counts and trends, in March 2020. Unique users, visits, and interactions with tool features were measured. In August 2020, a cross-sectional survey assessed respondent characteristics, COVID-19 knowledge, behaviors, and preferences. TWC COVID-19 hub averaged 1.97 million unique users with over 2.6 million visits daily and an average interaction time of 1.63 min. Respondents reported being knowledgeable about COVID-19 (92.3%) and knowing relevant safety precautions (90.9%). However, an average of 35.3% of respondents reported not increasing preventive practices across behaviors surveyed due to information about COVID-19. In conclusion, we find a free weather application delivered COVID-19 data to millions of Americans. Despite confidence in knowledge and best practices for prevention, over one-third of survey respondents did not increase practice of preventive behaviors due to information about COVID-19.

## Lay Summary

A coronavirus disease 2019 (COVID-19) digital information hub, which displayed the number of COVID-19 cases in someone’s geographic area and trends in cases, was created for The Weather Company (TWC) application. We examined the usage of this hub postdeployment in March 2020. We also explored users’ COVID-19 knowledge, behaviors, and what information might be beneficial to users of the hub with a survey in August 2020. Unique users, visits, and interactions with the tool were measured. We find the TWC COVID-19 hub has averaged 1.97 million unique users with over 2.6 million visits daily and an average interaction time of 1.63 min. Respondents reported being knowledgeable about COVID-19 (92.3%) and knowing relevant safety precautions (90.9%). However, an average of 35.3% of respondents reported not changing their practice of measures to prevent the spread of COVID-19. We find a free weather application delivered COVID-19 data to millions of Americans. Despite respondents reporting confidence in their knowledge of COVID-19 and best practices, over one-third of survey respondents on average did not increase practice of preventive behaviors due to information about COVID-19.

## INTRODUCTION

The global coronavirus disease 2019 (COVID-19) pandemic has resulted in over 53 million cases and the deaths of over 820 000 people in the United States alone and is now the third-leading cause of death in the United States.^1^^,^^2^ Beyond the morbidity and mortality of the disease, the pandemic has had a pervasive impact on almost all aspects of daily life including how individuals work, do business, and go to school, resulting in a wide variety of new consumer health information needs. People have sought COVID-19 data and information to make informed decisions for themselves and their families. Dashboards tracking COVID-19 case counts and deaths have proliferated since the beginning of the pandemic to inform the public.^3^ However, few studies have reported on usage of these tools among consumers and how they might address COVID-19 knowledge, needs, and behaviors. Therefore, an opportunity exists to understand the impact of COVID-19 data and information sharing and to inform public health messaging about what steps consumers should take to reduce spread of the disease.

The Weather Company (TWC) app for iOS and Android and weather.com website are consumer-facing tools that deliver free weather information to over 425 million users globally each month, providing 25 billion forecasts per day.^4^ As part of IBM’s corporate social responsibility initiatives in response to the pandemic, TWC provided COVID-19 information from the US Centers for Disease Control and Prevention and developed an interactive digital data and information hub (ie, dashboard) to display COVID-19 US county and state-level per capita case counts, deaths, and trends using data from the World Health Organization and multiple national, state, and local public health authorities.^5^

## OBJECTIVES

The objective of this brief communication is to examine the impact of an interactive COVID-19 data and information hub embedded in a weather application, which supports consumers in making decisions based on ambient conditions. We report usage of COVID-19 health information through this tool and assess COVID-19-related knowledge, behaviors, and information needs of TWC users who responded to a survey administered through the tool.

## MATERIALS AND METHODS

**Figure 1.**
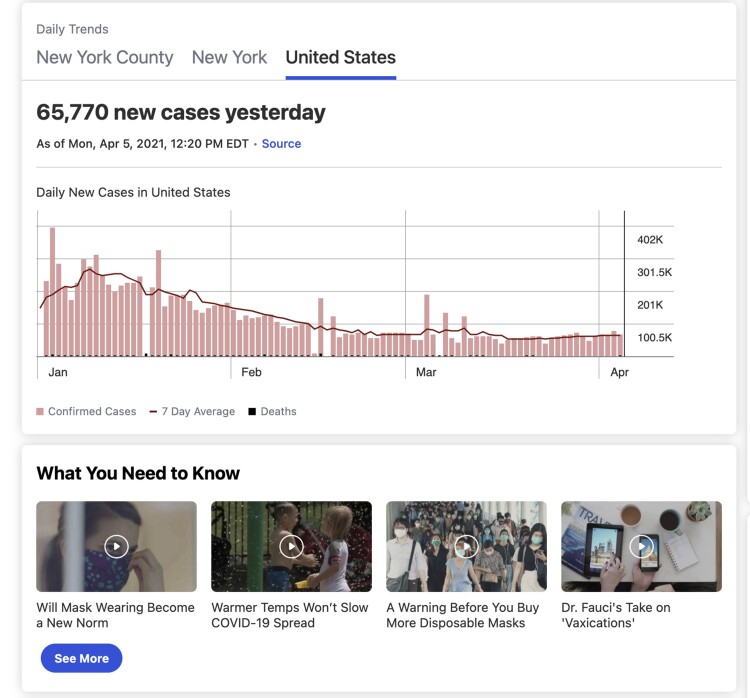
A sample view of the COVID-19 interactive hub (top) and COVID-19 information (bottom). Credit: weather.com, IBM.

The TWC COVID-19 hub provides an interactive “Incidents Map” of COVID-19 data and statistics, including confirmed cases and deaths by state and by county, where available, and trend graphs by state in the United States both in the web and app versions of TWC (see Figure 1). The latest information can be viewed for free at https://weather.com/coronavirus/ and through the app’s main tab. Case count data were collected daily from over 50 unique sources including free text PDFs, APIs, and interactive dashboards, and retrieved data went through a verification pipeline with automatic and manual processes. The automatic process examines the actual and projected rates of increase of case counts and deaths and flags issues when data are not following the predicted path. The manual verification process uses humans to audit the hub’s database with what is produced by county and state health departments on a weekly basis. In addition to case and death counts, news and information to help track the pandemic, as well as current public health information, are provided from trusted public health sources like the US Centers for Disease Control and Prevention (see Figure 1).

### Usage

The TWC COVID-19 hub launched on March 25, 2020 as a webpage that could be visited on nonmobile (desktop web browser) and mobile devices (through the TWC iOS or Android mobile apps and through mobile web browsers). Each day the following metrics were measured until study completion on August 12, 2020: (1) number of users (estimated using cookie-based traffic); (2) visits (defined as accessing the hub); (3) time spent per visit in seconds for app users; (4) number of interactions with the COVID-19 hub (defined as clicking on the interactive case or death counts); (5) number of interactions with COVID-19 hub trends (defined as clicking on the interactive trend line); (6) proportion of visits where a COVID-19 information video link was followed.

### Survey

From August 5, 2020 to August 12, 2020, we conducted a 12-question cross-sectional survey among all TWC app and website users who were US-based and 18 years of age or older. No rewards for completing the survey were provided to participants. For app users, the survey was presented as a pop-up screen with a link to an online survey; for web browser users the survey was presented as content to the right of the case count data display. COVID-19 knowledge, changes in practice of preventive behaviors, and information needs were assessed using Likert-scale response categories (see Supplementary Material for survey questions). Survey development was informed by the World Health Organization’s behavioral insights on COVID-19 survey tool.^6^ Demographic information including age, sex, education, living environment, race, and essential worker status were collected.

### Statistical analysis

Daily usage data, respondent sociodemographic characteristics, and survey responses were summarized with descriptive statistics. All analyses used R version 1.4.1103. The study was reviewed and determined exempt by the Western Institutional Review Board on June 22, 2020.

## RESULTS

### Usage

During the study period, the number of average daily unique users of the tool was 1 970 000. The hub had an estimated total of 367 044 641 visits (ie, times the hub was accessed) for an estimated average of 2 621 747 daily visits. Most visits were from the iOS app (58.56%), mobile web browsers (22.14%), followed by the Android app (15.78%), and then desktop visits (3.49%). The average time spent per visit by iOS and Android TWC app users was estimated to be 98 seconds. The estimated average number of interactions with the COVID-19 hub (defined as clicking on the interactive case or death counts) per day was 988 753 or 37.7% of average daily visits, whereas the average number of interactions (defined as clicking on the interactive trend line) with COVID-19 hub trends per day was 286 193 or 10.9% of average daily visits. 1.3% of visits followed a COVID-19 video content link.

### Survey demographics

Table 1 shows demographics of the 6972 survey respondents. Usage platforms included Android 36.8%, iOS 57.9%, mobile web browser 1%, and web browser 4.3%. Most users who responded to survey questions were White (90.5%), female (54.0%), with a mean age category of 60–69 years (31.6%). Most had a bachelor’s degree or higher (51.5%) and lived in rural (32.1%), suburban (51.2%), and urban (16.7%) settings. 28.6% were essential workers. User base demographics for TWC are available in the Supplementary Material.

**Table 1.** Demographics of survey respondents

Variable		Count (%)
Race (*N* = 6427)	White	5806 (90.3%)
	Black or African American	275 (4.3%)
	Other	200 (3.1%)
	Asian	71 (1.1%)
	Native Hawaiian or Pacific Islander	12 (0.2%)
	American Indian or Alaskan Native Students	63 (1.0%)
Ethnicity (*N* = 4723)	Hispanic	271 (5.7%)
	Non-Hispanic	4452 (94.3%)
Age (*N* = 6972)	18–29	259 (3.7%)
	30–39	403 (5.8%)
	40–49	821 (11.8%)
	50–59	1534 (22.0%)
	60–69	2205 (31.6%)
	70–79	1483 (21.3%)
	80+	267 (3.8%)
Sex (*N* = 6795)	Female	3671 (54.0%)
	Male	3124 (46.0%)
Education level (*N* = 6972)	Graduate degree	1717 (24.6%)
	Bachelor’s degree	1877 (26.9%)
	Some college	2233 (32.0%)
	High school	1058 (15.2%)
	Less than high school diploma	87 (1.3%)
Living environment (*N* = 6632)	Rural	2127 (32.1%)
	Suburban	3395 (51.2%)
	Urban	1110 (16.7%)
Essential worker status (*N* = 6972)	No	4977 (71.4%)
	Yes	1995 (28.6%)

### Survey results

Table 2 shows survey results. Most respondents rated their knowledge of how to prevent spread of novel coronavirus as “Very good” (57.2%) or “Good” (35.08%) and most said it was “Clear” (34.4%), or “Very clear” (56.5%) what safety precautions they should take to prevent COVID-19 spread. To stay informed about the pandemic, most respondents relied on websites or online news (69.2%), television (54.9%), and government health agencies (50.1%). As a result of information about COVID-19, participants had increased their practice of commonly recommended preventive behaviors to prevent the spread of the disease due to information about COVID-19^7^ including wearing a face mask (68.9%), physical distancing (68.6%), and hand washing for at least 20 seconds (68.1%). Among the features provided by TWC, respondents reported local data and statistics about novel coronavirus (64.4%) and news articles and videos about novel coronavirus (53.4%) were helpful. In the future, respondents were most interested in local information about hotspots of COVID-19 (72.7%), severity of cases (71.6%), and trends in cases from TWC (71.0%). The Supplementary Material contains complete and detailed results.

**Table 2.** Survey questions and results

	Variable		Count (%)
**Knowledge**	Knowledge about how to prevent COVID-19 (*N* = 6972)	Very good	3985 (57.2%)
		Good	2446 (35.1%)
		Fair	480 (6.9%)
		Poor	34 (0.5%)
		Very poor	27 (0.4%)
Perceived clarity	Perceived clarity of information on how to prevent COVID-19 (*N* = 6972)	Very clear	3937 (56.5%)
		Clear	2395 (34.4%)
		Neutral	320 (4.6%)
		Poor	124 (1.8%)
		Very poor	196 (2.8%)
Information sources	COVID-19 information sources used ‘Very often” or “Often”^a^	Online news websites (*N* = 6972)	4824 (69.2%)
		Television (*N* = 6972)	3824 (54.9%)
		Official government websites (*N* = 6972)	3493 (50.1%)
		Conversations with friends, family, colleagues *N* = 6972)	3382 (48.5%)
		TWC app/website or similar apps/websites (*N* = 6972)	2916 (41.8%)
		Daily or weekly newspapers (*N* = 6972)	1976 (28.3%)
		Social media (*N* = 6972)	1705 (24.5%)
		Radio (*N* = 6972)	1278 (18.3%)
Preventive behaviors	“Very much” or “Somewhat” increased preventative behaviors due to COVID-19 information^a^	Wearing a face mask (*N* = 6972)	4802 (68.9%)
		Physical distancing (*N* = 6972)	4784 (68.6%)
		Covering mouth when coughing (*N* = 6972)	4767 (68.4%)
		Hand washing for at least 20 s (*N* = 6972)	4751 (68.1%)
		Staying home when sick (*N* = 6972)	4556 (65.4%)
		Use of hand sanitizer (*N* = 6972)	4497 (64.5%)
		Disinfecting surfaces (*N* = 6972)	4339 (62.2%)
		Avoiding touching eyes, nose, and mouth (*N* = 6972)	4224 (60.6%)
		Self-isolation (*N* = 6972)	3848 (55.2%)
		Herbal supplements (*N* = 6972)	1291 (18.5%)
		Antibiotics (*N* = 6972)	1042 (14.9%)
TWC information	TWC COVID-19 information “Very much” or “Somewhat” helpful^a^	Local data and statistics about novel coronavirus (*N* = 6972)	4489 (64.4%)
		News articles and videos about novel coronavirus (*N* = 6972)	3762 (53.4%)
Interest in information topics	Respondents interested in COVID-19 topics from TWC^a^	Hotspots of local novel coronavirus (*N* = 6972)	5039 (72.7%)
		Severity of local cases of novel coronavirus (*N* = 6972)	4989 (71.6%)
		Trends in local novel coronavirus cases (*N* = 6972)	4949 (71.0%)
		When the outbreak of COVID-19 will end (*N* = 6972)	4759 (68.3%)
		Ways to prevent getting novel coronavirus (*N* = 6972)	4633 (66.5%)
		Where to get tested for novel coronavirus (*N* = 6972)	4442 (63.7%)
		Total local COVID-19 cases (*N* = 6972)	4436 (63.6%)
		Clinical trials related to novel coronavirus (*N* = 6972)	4017 (57.6%)

Percentage values do not add up to 100 because we report on positive Likert-scale responses for these questions. Full survey results are in the Supplementary Material.

COVID-19: coronavirus disease 2019; TWC: The Weather Company.

## DISCUSSION

Consumers use a wide variety of applications providing information sources about local conditions such as traffic, pollen levels, and weather to make informed decisions about daily activities. The COVID-19 pandemic affected nearly every aspect of daily life, and this study demonstrated avid engagement with a COVID-19 data and information hub delivered through a weather application. Almost 2 million unique users each day accessed the tool for a total of over 300 million visits, with each visit typically spanning almost 2 min. This illustrates how consumers sought local COVID-19 information from nontraditional sources during the pandemic to inform daily activities and behaviors.

At approximately 6 months after the pandemic onset and deployment of the tool, most respondents said they had good knowledge about how to prevent the spread of the disease and that it was clear what safety precautions they should take to prevent COVID-19 spread. At the same time, over one-third of respondents on average reported not increasing practicing preventive behaviors important for stopping the spread due to information about COVID-19. Although the majority of respondents practiced preventive behaviors, some reported practices that may actually be harmful, such as increasing their use of antibiotics. Therefore, there exists a continued need to provide information about COVID-19 that prompts appropriate actions to support the health of the general public.

Although consumer health information preferences are highly personal, they are not always predicted by sociodemographic characteristics.^8^ Although our survey received over 6000 responses, our survey participants reflect only a small percentage of TWC COVID-19 hub users, and may not represent the entire United States, especially when it comes to race, with over 90% of our respondents identifying as White and over half (51.5%) having a Bachelor’s or Graduate degree. Our survey respondents were similar to the TWC user base in terms of demographics, which may be indicative of the growing digital divide and should be further explored. Future research should try to understand how to better engage underrepresented subpopulations of TWC users. This is especially relevant given the current need to combat misinformation and encourage vaccination, especially among specific demographic groups.

## CONCLUSION

We find that during the COVID-19 pandemic almost 2 million American each day sought information of the novel disease from a free weather application. Through a survey among a subset of 6972 users, we find respondents were confident in their knowledge about COVID-19 and how to prevent its spread but a large proportion (35.3%) on average did not report that information about COVID-19 had impacted their practice of preventive behaviors. Continued research is needed to understand how best to provide public health information about COVID-19 that prompts appropriate public health-related actions.

## AUTHOR CONTRIBUTIONS

MM contributed to the conception of the work; the acquisition, analysis, and interpretation of data; the drafting of the work; and critical revision. CV contributed to the conception of the work and the acquisition of the data. CB contributed to the conception of the work; the acquisition, analysis and interpretation of data, and critical revision. WF contributed to the acquisition of the data. RH contributed to the acquisition of the data. GPJ contributed to the conception of the work; the interpretation of data; the drafting of the work; and critical revision.

## SUPPLEMENTARY MATERIAL

Supplementary material is available at *JAMIA Open* online.

## FUNDING

This study is funded by IBM Watson Health.

## CONFLICT OF INTEREST STATEMENT

The authors of this study are employed by IBM Watson Health and The Weather Company.

## DATA AVAILABILITY

The data underlying this article will be shared on reasonable request to the corresponding author.

## Supplementary Material

ooac016_Supplementary_Data
