# Supplementary material for: Assessing and addressing COVID-19 information needs via a weather application
Source: JAMIA Open. 2022 Mar 26;5(1):ooac016. doi: 10.1093/jamiaopen/ooac016 (PMC9037469; doi:10.1093/jamiaopen/ooac016)
Supplement: ooac016_Supplementary_Data [file ooac016_supplementary_data.docx]

Supplement to:

“Assessing and Addressing COVID-19 Information Needs via a Weather Application”

Table of Contents

**Survey questions**………………………………………………………………………………………………………page 2

**All survey results**………………………………………………………………………………………………………page 6

**The Weather Company User Base Demographics**……………………………………………………page 10

**Survey Questions**

| **Knowledge about behavior** | | |
| --- | --- | --- |
| **1** | How would you rate your knowledge level on how to prevent spread of COVID -19? | \| Very poor \| Poor \| Fair \| Good \| Very good \| \| --- \| --- \| --- \| --- \| --- \| \| **〇** \| **〇** \| **〇** \| **〇** \| **〇** \| |
| **2** | Do you feel it is clear what safety precautions you should be taking to prevent spread of COVID -19? | \| Very unclear \| Unclear \| Neither clear or unclear \| Clear \| Very clear \| \| --- \| --- \| --- \| --- \| --- \| \| **〇** \| **〇** \| **〇** \| **〇** \| **〇** \| |

| **Information about the virus** | | |
| --- | --- | --- |
| **3** | How often do you use the following sources of information to stay informed about the novel coronavirus? | \|  \| **Never** \| **Rarely** \| **Sometimes** \| **Often** \| **Very often** \| \| --- \| --- \| --- \| --- \| --- \| --- \| \| Television \|  \|  \|  \|  \|  \| \| Daily or weekly newspapers \|  \|  \|  \|  \|  \| \| Conversations with family, friend, or colleagues \|  \|  \|  \|  \|  \| \| Websites or online news pages \|  \|  \|  \|  \|  \| \| Social media (e.g. Facebook, Twitter, YouTube) \|  \|  \|  \|  \|  \| \| TWC app/website or similar apps/websites \|  \|  \|  \|  \|  \| \| Radio stations \|  \|  \|  \|  \|  \| \| Official, government press releases or county / state health agency websites \|  \|  \|  \|  \|  \| \| Other sources, namely: _____ [Free text] \|  \|  \|  \|  \|  \| |

**Survey Questions (Continued)**

| **Behaviors Practiced** | | |
| --- | --- | --- |
| **4** | Do you feel you have increased your practice of any of these preventative measures to stop infection from the novel coronavirus as a result of information provided by The Weather Company? | \|  \| **Not at all** \| **Not much** \| **Neutral** \| **Somewhat** \| **Very**  **much** \| \| --- \| --- \| --- \| --- \| --- \| --- \| \| Hand washing for at least 20 seconds \|  \|  \|  \|  \|  \| \| Avoiding touching your eyes, nose, and mouth \|  \|  \|  \|  \|  \| \| Use of hand sanitizer \|  \|  \|  \|  \|  \| \| Staying home when sick \|  \|  \|  \|  \|  \| \| Herbal supplements \|  \|  \|  \|  \|  \| \| Covering mouth when coughing \|  \|  \|  \|  \|  \| \| Wearing a face mask \|  \|  \|  \|  \|  \| \| Using antibiotics \|  \|  \|  \|  \|  \| \| Physical distancing (keeping minimum 6 feet between you and other persons outside your household) \|  \|  \|  \|  \|  \| \| Self-isolation \|  \|  \|  \|  \|  \| \| Disinfecting surfaces \|  \|  \|  \|  \|  \| \| Another preventive measure, please specify:______ \|  \|  \|  \|  \|  \| |

**Survey Questions (Continued)**

| **Information needs** | | | | |
| --- | --- | --- | --- | --- |
| **5** | Are either of the following features provided by The Weather Company about the novel corona virus helpful to you? | \|  \| **Not at all** \| **Not much** \| **Neutral** \| **Somewhat** \| **Very**  **much** \| \| --- \| --- \| --- \| --- \| --- \| --- \| \| News articles and videos about novel coronavirus \|  \|  \|  \|  \|  \| \| Local data and statistics about novel  coronavirus \|  \|  \|  \|  \|  \| | | |
| **6** | Would you like The Weather Company to provide more information or updates about any of the following: | \|  \| **Not at All Interested** \| **Not Very Interested** \| **Neutral** \| **Somewhat Interested** \| **Very**  **Interested** \| \| --- \| --- \| --- \| --- \| --- \| --- \| \| Total local COVID-19 cases \|  \|  \|  \|  \|  \| \| Hotspots of local novel coronavirus \|  \|  \|  \|  \|  \| \| Severity of local cases of novel coronavirus \|  \|  \|  \|  \|  \| \| Trends in local novel coronavirus cases \|  \|  \|  \|  \|  \| \| When the outbreak of COVID-19 will end \|  \|  \|  \|  \|  \| \| Where to get tested for novel coronavirus \|  \|  \|  \|  \|  \| \| Ways to prevent getting novel coronavirus \|  \|  \|  \|  \|  \| \| Clinical trials related to novel coronavirus \|  \|  \|  \|  \|  \| \| Other information or updates, please specify: ______ \|  \|  \|  \|  \|  \| | | |
| **Survey Questions (Continued)** | | | |  |
| **Demographics** | | | | |
| **7** | What is your age? | \| 18-29 \| 30-39 \| 40-49 \| 50-59 \| 60-69 \| 70-79 \| 80+ \| \| --- \| --- \| --- \| --- \| --- \| --- \| --- \| \| **〇** \| **〇** \| **〇** \| **〇** \| **〇** \| **〇** \| **〇** \| | | |
| **8** | What is your sex? | \| Male \| Female \| Prefer not to say \| \| --- \| --- \| --- \| \| **〇** \| **〇** \| **〇** \| | | |
| **9** | What is your living environment? | \| Rural \| Urban \| Suburban \| I’m not sure \| \| --- \| --- \| --- \| --- \| \| **〇** \| **〇** \| **〇** \| **〇** \| | | |
| **10** | What is your highest education level? | \| Less than high school diploma \| high school \| Some college \| Bachelor’s degree \| Graduate degree \| \| --- \| --- \| --- \| --- \| --- \| \| **〇** \| **〇** \| **〇** \| **〇** \| **〇** \| | | |
| **11** | What is your Race / Ethnicity? | \| **Race** \|  \| \| --- \| --- \| \| White \| **〇** \| \| Black or African American \| **〇** \| \| American Indian or Alaskan Native \| **〇** \| \| Asian \| **〇** \| \| Native Hawaiian or other Pacific Islander \| **〇** \| \| Other \| **〇** \| \|  \|  \| \|  \|  \| \|  \|  \| \|  \|  \| \|  \|  \| \|  \|  \| \|  \|  \| \|  \|  \| | \| **Ethnicity** \|  \| \| --- \| --- \| \| Not of Hispanic, Latino, or Spanish origin \| **〇** \| \| Hispanic, Latino, or Spanish origin \| **〇** \| \| Other \| **〇** \| \|  \|  \| \|  \|  \| \|  \|  \| | |
| **12** | Are you an essential worker? For information about job categories that are essential [click here](https://www.cdc.gov/coronavirus/2019-ncov/community/critical-workers/implementing-safety-practices.html) | \| Yes \| No \| \| --- \| --- \| \| **〇** \| **〇** \| | | |

**Survey Results**

| Variable | | Count (%) |
| --- | --- | --- |
| Knowledge about how to prevent COVID-19 | Very good  Good  Fair  Poor  Very poor | 3985 (57.2%)  2446 (35.1%)  480 (6.9%)  34 (0.5%)  27 (0.4%) |
| Perceived clarity of information on how to prevent COVID-19 | Very clear  Clear  Neutral  Poor  Very poor | 3937 (56.5%)  2395 (34.4%)  320 (4.6%)  124 (1.8%)  196 (2.8%) |
| COVID-19 information sources (Online news websites) | Never  Often  Rarely  Sometimes  Very often | 342 (4.9%)  2339 (33.6%)  437 (6.3%)  1369 (19.6%)  2485 (35.6%) |
| COVID-19 information sources (Television) | Never  Often  Rarely  Sometimes  Very often | 860 (12.3%)  1899 (27.2%)  859 (12.3%)  1429 (20.5%)  1925 (27.6%) |
| COVID-19 information sources (Official government websites) | Never  Often  Rarely  Sometimes  Very often | 522 (7.50%)  2113 (30.3%)  829 (11.9%)  2126 (30.5%)  1382 (19.8%) |
| COVID-19 information sources (Conversations with friends, family, colleagues) | Never  Often  Rarely  Sometimes  Very often | 298 (4.3%)  2118 (30.4%)  808 (11.6%)  2484 (35.6%)  1264 (18.1%) |
| COVID-19 information sources (TWC app/website or similar apps/websites) | Never  Often  Rarely  Sometimes  Very often | 1236 (17.7%)  1485 (21.3%)  1046 (15%)  1774 (25.4%)  1431 (20.52%) |
| COVID-19 information sources (Daily or weekly newspapers) | Never  Often  Rarely  Sometimes  Very often | 2566 (36.8%)  1025 (14.7%)  1206 (17.3%)  1224 (17.6%)  951 (13.6%) |

**Survey Results (Continued)**

| Variable | | Count (%) |
| --- | --- | --- |
| COVID-19 information sources (Social media) | Never  Often  Rarely  Sometimes  Very often | 2279 (32.7%)  917 (13.2%)  1475 (21.2%)  1513 (21.7%)  788 (11.3%) |
| COVID-19 information sources (Radio) | Never  Often  Rarely  Sometimes  Very often | 2274 (32.6%)  861 (12.4%)  1664 (23.9%)  1756 (25.2%)  417 (6.0%) |
| Increased preventative behaviors due to COVID-19 information (Wearing a face mask) | Neutral  Not at all  Not much  Somewhat  Very much | 511 (7.3%)  1377 (19.8%)  282 (4.0%)  810 (11.6%)  3992 (57.3%) |
| Increased preventative behaviors due to COVID-19 information (Physical distancing) | Neutral  Not at all  Not much  Somewhat  Very much | 576 (8.3%)  1343 (19.3%)  269 (3.9%)  1169 (16.8%)  3615 (51.9%) |
| Increased preventative behaviors due to COVID-19 information (Covering mouth when coughing) | Neutral  Not at all  Not much  Somewhat  Very much | 652 (9.4%)  1365 (19.6%)  188 (2.7%)  635 (9.1%)  4132 (59.3%) |
| Increased preventative behaviors due to COVID-19 information (Staying home when sick) | Neutral  Not at all  Not much  Somewhat  Very much | 826 (11.9%)  1393 (20.0%)  197 (2.8%)  750 (10.8%)  3806 (54.6%) |
| Increased preventative behaviors due to COVID-19 information (Use of hand sanitizer) | Neutral  Not at all  Not much  Somewhat  Very much | 677 (9.7%)  1485 (21.3%)  313 (4.5%)  1183 (17.0%)  3314 (47.5%) |
| Increased preventative behaviors due to COVID-19 information (Self-isolation) | Neutral  Not at all  Not much  Somewhat  Very much | 1030 (14.8%)  1703 (24.4%)  391 (5.6%)  1655 (23.8%)  2193 (31.5%) |

**Survey Results (Continued)**

| Variable | | Count (%) |
| --- | --- | --- |
| Increased preventative behaviors due to COVID-19 information (Hand washing for at least 20 seconds) | Neutral  Not at all  Not much  Somewhat  Very much | 619 (8.9%)  1337(19.2%)  265 (3.8%)  1188 (17.0%)  3563 (51.1%) |
| Increased preventative behaviors due to COVID-19 information (Disinfecting surfaces) | Neutral  Not at all  Not much  Somewhat  Very much | 818 (11.7%)  1450 (20.8%)  365 (5.2%)  1663 (23.9%)  2676 (38.4%) |
| Increased preventative behaviors due to COVID-19 information (Avoiding touching eyes, nose, and mouth) | Neutral  Not at all  Not much  Somewhat  Very much | 880 (12.6%)  1442 (20.7%)  426 (6.1%)  1698 (24.4%)  2526 (36.2%) |
| Increased preventative behaviors due to COVID-19 information (Antibiotics) | Neutral  Not at all  Not much  Somewhat  Very much | 1268 (18.2%)  4095 (58.7%)  567 (8.1%)  581 (8.3%)  461 (6.6%) |
| TWC COVID-19 information (Local data & statistics about novel coronavirus) | Neutral  Not at all helpful  Not very helpful  Somewhat helpful  Very helpful | 840 (12.1%)  1168 (16.8%)  475 (6.8%)  1868 (26.8%)  2621 (37.6%) |
| TWC COVID-19 information (News articles & videos about novel coronavirus) | Neutral  Not at all helpful  Not very helpful  Somewhat helpful  Very helpful | 1158 (16.6%)  1413 (20.3%)  639 (9.2%)  2171 (31.1%)  1591 (22.8%) |
| Respondents interested in COVID-19 topics from TWC (Hotspots of local novel coronavirus) | Neutral  Not at all interested  Not very interested  Somewhat interested  Very interested | 643 (9.2%)  1004 (14.4%)  286 (4.1%)  1361 (19.5%)  3678 (52.8%) |

| Survey Results (Continued) | | |
| --- | --- | --- |
| Variable | | Count (%) |
| Respondents interested in COVID-19 topics from TWC (Severity of local cases of novel coronavirus) | Neutral  Not at all interested  Not very interested  Somewhat interested  Very interested | 713 (10.2%)  992 (14.2%)  278 (4.0%)  1416 (20.3%)  3573 (51.3%) |
| Respondents interested in COVID-19 topics from TWC (Trends in local novel coronavirus cases) | Neutral  Not at all interested  Not very interested  Somewhat interested  Very interested | 733 (10.5%)  1005 (14.4%)  284 (4.1%)  1496 (21.5%)  3454 (49.5%) |
| Respondents interested in COVID-19 topics from TWC (When the outbreak of COVID-19 will end) | Neutral  Not at all interested  Not very interested  Somewhat interested  Very interested | 901 (12.3%)  1028 (14.8%)  284 (4.1%)  1223 (17.5%)  3536 (50.7%) |
| Respondents interested in COVID-19 topics from TWC (Ways to prevent getting novel coronavirus) | Neutral  Not at all interested  Not very interested  Somewhat interested  Very interested | 1001 (14.4%)  1064 (15.3%)  274 (4.0%)  1282 (18.4%)  3351 (48.1%) |
| Respondents interested in COVID-19 topics from TWC (Where to get tested for novel coronavirus) | Neutral  Not at all interested  Not very interested  Somewhat interested  Very interested | 1048 (15.0%)  1123 (16.1%)  359 (5.2%)  1519 (21.8%)  2923 (41.9%) |
| Respondents interested in COVID-19 topics from TWC (Total local COVID-19 cases) | Neutral  Not at all interested  Not very interested  Somewhat interested  Very interested | 994 (14.3%)  1113 (16.0%)  429 (6.2%)  1498 (21.5%)  2938 (42.1%) |
| Respondents interested in COVID-19 topics from TWC (Clinical trials related to novel coronavirus) | Neutral  Not at all interested  Not very interested  Somewhat interested  Very interested | 1237 (17.7%)  1254 (18.0%)  464 (6.7%)  1566 (22.5%)  2451 (35.2%) |

**The Weather Company User Base Demographics**

|  | **The Weather Channel (Mobile App)*** | **Weather.com (Desktop)*** |  |
| --- | --- | --- | --- |
| Hispanic/Spanish Origin Descent | 9% | 11% |  |
| White/Caucasian | 82% | 69% |  |
| Black/African American | 7% | 6% |  |
| American Indian/Alaska Native | 1% | 1% |  |
| Asian/Native Hawaiian/Pacific Islander | 7% | 8% |  |
| Other race | 2% | 22% |  |
| 18-24 yrs old | 6% | 6% |  |
| 25-34 yrs old | 15% | 18% |  |
| 35-44 yrs old | 15% | 19% |  |
| 45-54 yrs old | 19% | 19% |  |
| 55-64 yrs old | 24% | 19% |  |
| 65+ yrs old | 21% | 19% |  |
| Male | 52% | 56% |  |
| Female | 48% | 44% |  |
| Less than High School (edu level) | 0.40% | N/A** |  |
| Some High School (edu level) | 1.80% | 6% |  |
| Completed High School or Equivalent (edu level) | 14% | 28% |  |
| Some College (edu level) | 20% | 18% |  |
| Associates Degree (edu level) | 11% | 14% |  |
| Bachelors Degree (edu level) | 32% | 24% |  |
| Post Graduate Degree (edu level) | 21% | 13% |  |
|  |  |  |  |
| Citation: Comscore Plan Metrix® Audience Profile, United States, Desktop-Only - WEATHER.COM, and Mobile - The Weather Channel (Mobile App), July-September 2020 avg, % Composition Unique Visitors. | | | |
|  |  |  |  |
| * Comscore uses a proprietary methodology to calculate audience overlaps across desktop and mobile for a target web entity. Some category totals may not equal 100%. | | | |
| ** Data not available |  |  |  |
